# Alterations of the Danger Zone after Preparation of Curved Root Canals Using WaveOne with Reverse Rotation or Reciprocation Movements

**DOI:** 10.7508/iej.2015.03.002

**Published:** 2015-07-01

**Authors:** Yazdan Shantiaee, Omid Dianat, Payam Paymanpour, Golnaz Nahvi, Mohammad Ali Ketabi, Golbarg Kolahi Ahari

**Affiliations:** a *Iranian Center for Endodontic Research, Research Institute of Dental sciences, Department of Endodontics, Dental School, Shahid Beheshti University of Medical Sciences, Tehran, Iran; *; b *DentalStudent, Dental School, Shahid Beheshti University of Medical Sciences, Tehran, Iran; *; c * Department of Endodontics, Dental School, Aja University of Medical Sciences, Tehran, Iran;*; d * Endocrinology and Metabolism Research Center, Medical School, Shahid Beheshti University of Medical Sciences, Tehran, Iran*

**Keywords:** Danger Zone, Reciprocating Handpiece, Reciprocation, Rotation, WaveOne

## Abstract

**Introduction:**

The aim of this study was to compare the changes that occur in the danger zone (DZ) after preparation of curved mesiobuccal (MB) canals of mandibular first molars with WaveOne instruments in two different movements [reciprocation (RCP) and counter-clockwise rotation (CCWR)] by means of cone-beam computed tomography (CBCT).

**Methods and Materials:**

MB canals of 30 mandibular molars were randomly divided into 2 groups (*n*=15); WaveOne/RCP and WaveOne/CCWR. Pre- and post-instrumentation CBCT images were assessed for changes in the dentin thickness in DZ (2 and 4 mm below the highest point of the root furcation) in both groups. Data was analyzed using the repeated measures ANOVA test.

**Results:**

There was no statistically significant difference between two experimental groups in terms of remaining dentin thickness at 2 and 4 mm levels below the highest point of the furcation (*P*>0.05).

**Conclusion:**

The efficacy of WaveOne instrument on changes of the dentin thickness in the DZ was not affected by different file movements.

## Introduction

It is generally accepted that the strength of endodontically treated roots is directly dependent on the amount of remaining dentin. Aggressive removal of dentin can potentially weaken the root structure that consequently leads to root fracture or strip perforation especially in high risk areas such as danger zone (DZ) (the furcal side of the root canal wall) which is highly vulnerable to stripping by injudicious filing [1-3]. The thickness of the DZ could be analyzed using different techniques, some of which have disadvantages such as being expensive, time-consuming or detrimental to specimens. Cone-beam computed tomography (CBCT) is a practical nondestructive technique for assessment of the exact location and anatomy of the root canals before and after shaping [4].

Not all root canals are straight and when obtuse curvatures are present, endodontic preparation becomes more challenging; all available preparation techniques have the tendency to alter the original shape of the canal to different extents [5]. Therefore evaluating the ability of a given instrumentation technique in maintaining the original canal shape is necessary, especially in curved root canals. 

Aiming at preserving the root canal curvatures, the balanced forced technique was proposed by Roane *et al.* [6]. Recently, this technique has again become the center of interest as the origin of the reciprocal movement of single-file engine driven systems. Reciprocation (RCP) motion includes several back-and-forth movements with different degrees, which may impact the performance and resistance to fracture of nickel-titanium (NiTi) instruments [7]. 

**Figure 1:**
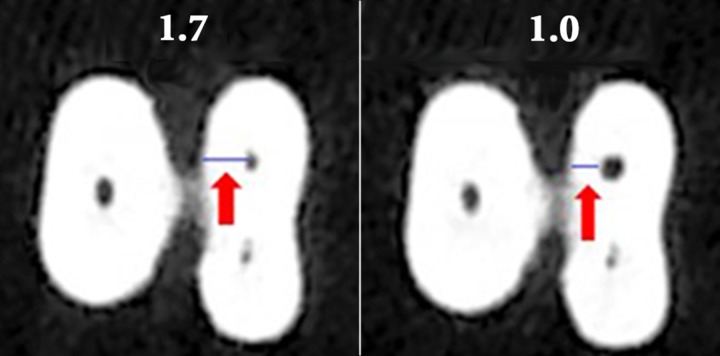
Dentin thickness measured in the danger zone area in CBCT images obtained before (left) and after (right) preparation

WaveOne (Dentsply Maillefer, Ballaigues, Switzerland) is amongst these systems that employs RCP movements. A large (170^°^) counter-clockwise rotation (CCWR) engages the instrument cutting edges to dentin so that it cuts dentin and penetrates in the canal; whereas a smaller rotation angle in the clockwise rotation (CWR) (50^°^) allows immediate file disengagement and its safely moving along the canal path [8]. RCP motion is claimed to reduce the screwing effect and instrument separation [9]. On the other hand it is said that due to the force applied to the apical portion during pecking of the reciprocating instrument, formation of detrimental microcracks are likely [2]. Although according to the manufacturer, WaveOne should be used in 170^°^ CCWR and 50^°^ CWR motion, there is no evidence that the recommended manner is also the best one. On the other hand, there are some single-files systems that employ full rotation instead of RCP [OneShape (Micro Méga, Besançon, France) or F360 (Brasseler, Lemgo, Germany)] that can be installed on current common electric motors. A weak point of reciprocal systems is the high initial cost due to the need for extra equipment (not all electric torque-controlled motors support the back-and-forth movements) [10]. 

The aim of this *in vitro* investigation was to compare the ability of WaveOne instruments with a CCWR movement to similar instruments with RCP motion in maintaining the thickness of DZ in curved root canals using CBCT imaging system.

## Materials and Methods

Using sample size calculation menu of Minitab, and considering *α*=0.5 and *β*=0.1, the minimum estimated sample size for each group was estimated to be 15. In this *in vitro* study thirty mesiobuccal (MB) roots of mandibular first molars that were extracted due to periodontal disease, were used. Immediately after extraction, all soft tissues and calculi were removed and radiographs were taken to select the teeth with mature apices and free of any resorption, calcification or previous endodontic obturation.

Before use, the teeth were decontaminated by immersion in 5.25% sodium hypochlorite (NaOCl) (Golrang, Pakshoo, Tehran, Iran) for 30 min. Teeth were then stored in sterile normal saline (Samen Co., Tehran, Iran) at room temperature. 

The storage time of all teeth was less than 2 months before initiation of the experiment. All canals were negotiated with a #15 K-file (Dentsply, Maillefer, Ballaigues, Switzerland) in order to verify the orientation of the canal axis and the absence of obstructions.

To determine the radii and degrees of curvature, digital periapical radiographs were taken from each tooth from buccal, mesial and distal aspects using a charge coupled device (CCD) sensor (Dr. Suni, Suni Medical Imaging, San Jose, CA, USA). MB roots with severe angle of curvature (20-45 degrees as described by Schneider [11]) were selected. The roots were mounted using a polyvinyl siloxane impression material (Speedex; Coltene AG, Alstatten, Switzerland) on a custom made mounting jig (2×6×6 cm) which served as a stable guide to take the post instrumentation images of the samples with kVp= 110, mA= 29.39, exposure time= 5.4 sec, voxel size= 0.100×0.100×0.100 mm, axial thickness= 0.100 and field of view (FOV)=6×6 cm set in Denture Scan mode. Dimensions of the jig matched the FOV of the NewTom VGI 9000 CBCT device (QR SRL Co., Verona, Italy). The coronal portions of the teeth were embedded in polyvinyl siloxane impression material, leaving the roots oriented upward; the highest point of the furcation area was determined as the reference point using a guiding radiopaque pin. To compare the dentin thickness in the cervical third of the roots in the DZ, 0.5-mm thick cross-sectional axial CBCT images were acquired before and after instrumentation. Images were taken from 2 and 4-mm areas below the reference point. The beginning and the end point of the scanning (on the Z axis) were recorded to allow repeated scanning of the specimen at similar horizontal levels.

The crowns of the teeth were maintained to stimulate the clinical practice. The teeth were randomly allocated to two identical groups of 15 (*n*=15); in each group 5 canals were considered as control that were left uninstrumented. For the test groups WaveOne primary instruments (25/0.08) were used in RCP or CCWR motion.

The working length (WL) was determined by reducing 1 mm from the length of a #15 K-file emerging at the apical foramen. Glide path was prepared using #15 K-file in both groups. Each canal was filled with 5.25% NaOCl as lubricant and shaped with WaveOne files until reaching the WL. Teeth in group RCP were prepared by instruments installed on a gear reduction handpiece powered by a torque-controlled motor (X-Smart plus reciprocating endodontic motor, Dentsply Maillefer, Ballaigues, Switzerland) set on reciprocal mode. Files were used in slow in-and-out pecking motion. The flutes of the instruments were cleaned after three pecks. Each file was used to 

**Figure 2:**
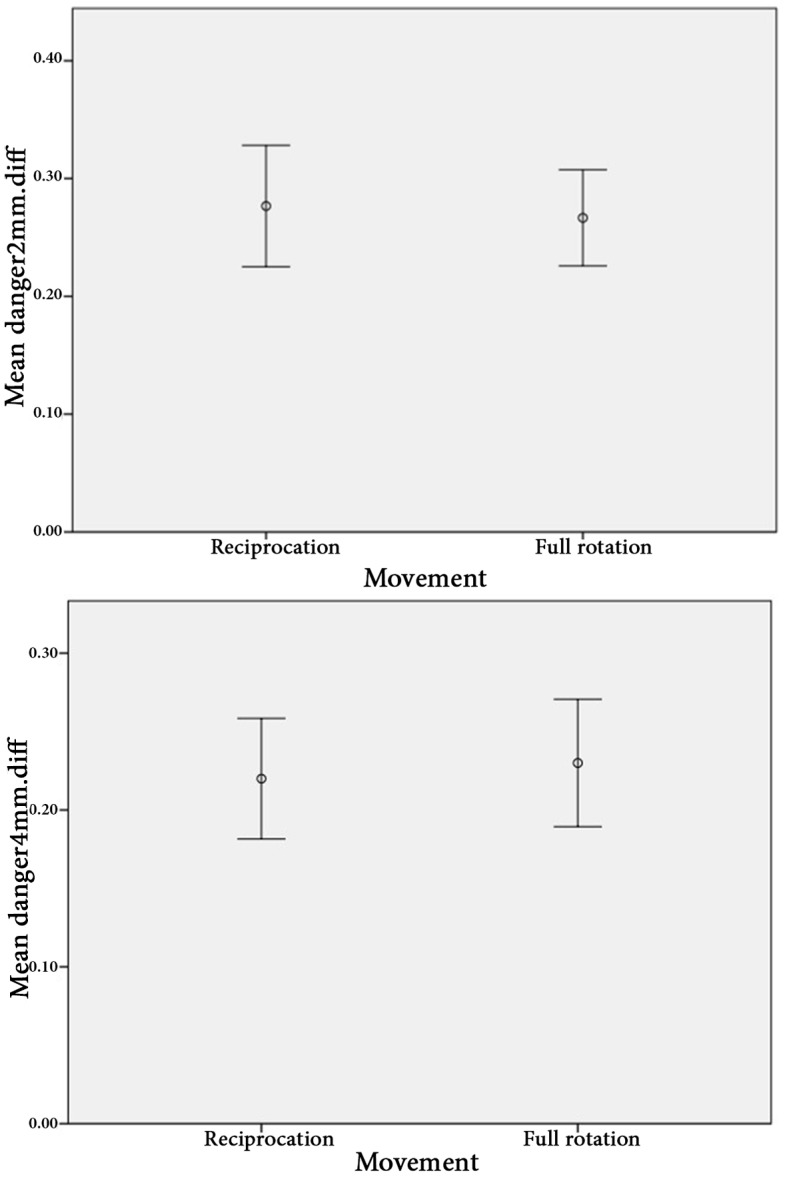
The error bar of the mean values of changes in the dentin thickness in danger zone in 2 and 4 mm sections [confidence interval (CI)=95%]

prepare 4 canals and the preparation time was recorded. In group CCWR teeth were instrumented by using WaveOne files installed on the same device which was set in continuous reverse rotation at speed of 300 rpm and the torque of 5 N/cm. For both groups during and after the use of each file, canals were irrigated with 5 mL of a 5.25% NaOCl solution by using a 30-gauge needle (Monoject; Sherwood Medical, St. Louis, MO, USA). 

The specimens including test and control samples were then replaced at the same position on the jig and then were scanned under the similar conditions. Assessment of scans was done by the recommended software, NTT Viewer version 3.00 (NTT Software Corporation, Yokohama, Japan). *MPR* Screen was utilized for measuring. The *Zoom* tool was applied to allow a better visualization of the teeth. The vertical and horizontal bars were used as reference for alignment of the images. The *Distance* tool (on coronal section) was employed to determine the measure from the highest point of the furcation area up to 2- and 4-mm distances apically. Then the horizontal bar was adjusted 2 and 4 mm from furcation area, generating an image in the axial section. Thickness of the canal wall was measured within the axial plane at two specified locations. To evaluate the alterations in dentin thickness, the shortest distance from the inner canal wall to the corresponding outer wall of the canal (mesial and distal) in uninstrumented and instrumented canals were measured in both safe zone and DZ of the aforementioned sections (Figure 1). 

**Figure 3:**
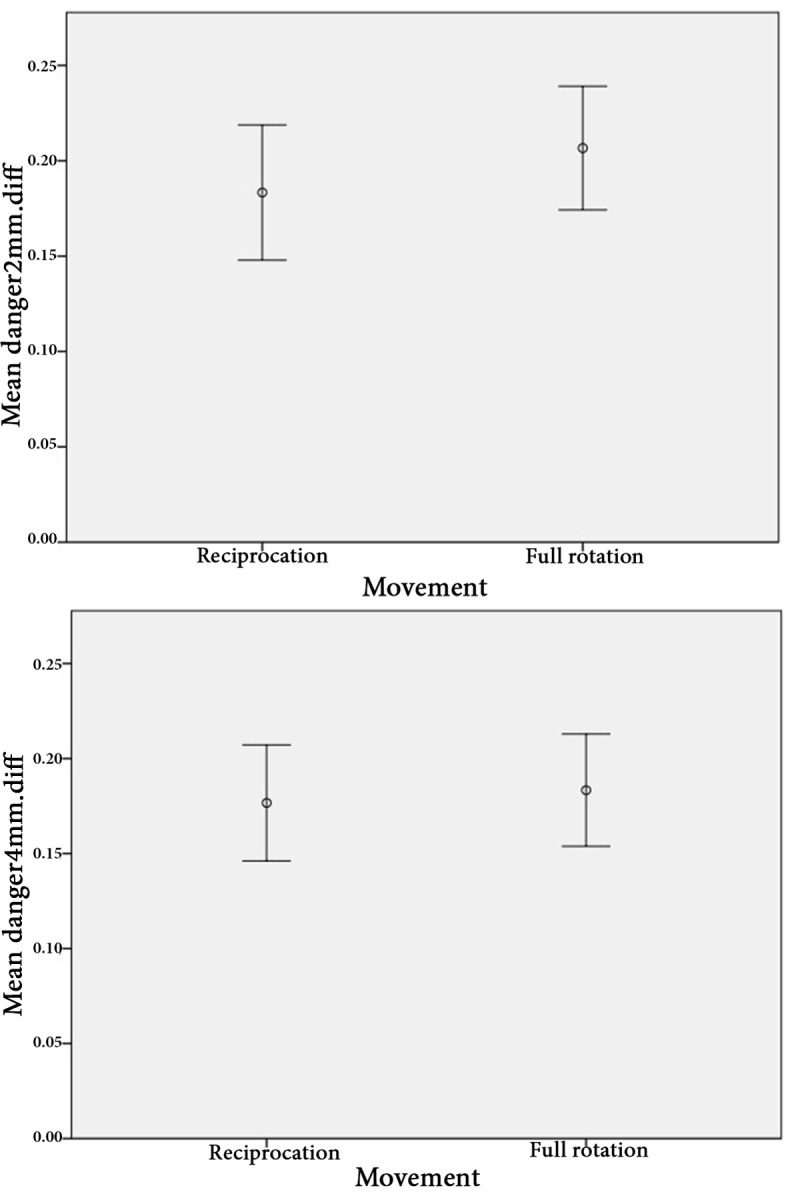
The error bar of the mean values of changes in the dentin thickness in safety zone in 2 and 4 mm sections [confidence interval (CI)=95%]

The distribution of the obtained data was analyzed by One-Sample Kolmogorov-Smirnov test. This test showed that the data points did not pass the normality test and the distribution of the data obtained by this study did not follow a Gaussian pattern. The mean changes of dentin thickness and the comparison between groups was carried out using repeated measures ANOVA test (*P*<0.05).

## Results

The results showed that the remaining dentin thickness in both instrumentation techniques was not significantly different in two sections (2 and 4 mm apical to the furcation highest point) both in DZ and safety zone (*P*>0.05) (Figures 2 and 3 and Table 1). No instrument fracture was reported in this study.

## Discussion

The current study investigated the changes of dentin thickness in danger zone in severely curved MB canals of mandibular first molars in two different movements (RCP and full CCWR) of WaveOne instruments. The result demonstrated that the type of movement did not affect the amount of remained dentin.

According to the manufacturer’s protocol, WaveOne single file system is designed specifically to be used in RCP motion. The results of previous studies on simulated canals in resin blocks showed that RCP motion decreases the risk of strip perforation in the curved canals in comparison with full rotation [12]. One explanation may be the type of motion. However, the variable cross sectional design, the reverse cutting edges and the M-Wire NiTi alloy used for manufacturing the file may have role.

Nevertheless, there are some single file systems that apply full rotational movement and are also able to preserve the original shape of the canal [13] and offer the advantage of being applicable with routine electric torque-controlled motors. More investigations are needed to explain if the result is caused by the type of file movement or other factors such as cross sectional design, alloys used in manufacturing the file and *etc*.

In some recent studies, mechanical properties including cyclic fatigue life and resistance to flexural fatigue of reciprocating files were evaluated. Recent literature shows that RCP motion can extend the cyclic fatigue life in comparison with continuous rotation [14, 15], but there is no study that exclusively deals with the effect of motion type on changes in the dentin thickness in canals prepared by single-files systems. Further investigation is required to determine the optimal RCP speed and angle that maintains the acceptable remaining dentin thickness.

**Table1:** Mean±SD of dentin thickness changes in different zones

	**Zone **	**Mean (SD)**	
		**RCP**	**CCWR**
**2-mm section**	**Danger zone**	0.26 (0.14)	0.28 (0.13)
	**Safety zone**	0.16 (0.08)	0.19 (0.07)
**4-mm section**	**Danger zone**	0.24 (0.10)	0.22 (0.12)
	**Safety zone**	0.17 (0.08)	0.16 (0.08)

In the current study the thickness of canal wall was measured before and after preparation at 2 and 4 mm below the furcation; because according to the results of previous studies the distal wall was reduced to the largest extent at that level [16]. The concept of setting the furcation area as a reference point was adopted based on a pioneer research stating that this area of the canal is more prone to perforations [1]. In one study, the distance from the root canal wall to the root surface was measured only once after root canal instrumentation. So, there would be no reliable basis for evaluating dentin thickness changes after canal preparation [17]. 

In some studies, simulated root canals in resin blocks were utilized to evaluate the shaping ability of the instruments. Resin blocks allow standardization and avoid the effect of anatomic variables of natural teeth samples such as size, shape and also the degree, taper, location and radius of curvature. However, they do not duplicate the real action of the instruments in the root canals of natural teeth. The hardness of plastic materials does not resemble that of dentin [8, 18]. Another disadvantage is heat generation, which softens the resin material and leads to binding of cutting blades or may lead to instrument separation [19]. Therefore, this study was conducted on natural extracted human teeth which provide conditions close to clinical situation.

Several methodologies have been proposed to assess the effect of root canal preparation with different instruments on the thickness of dentin wall such as microscopic analyses [20], silicone impressions [21], muffle system [16], scanning electron microscopy (SEM) [22], histologic sections [23], serial sectioning [24], endodontic cubes [25], radiographic comparisons [26], multislice spiral computed tomography (CT) scanner [27], CBCT [28, 29] and micro-computed tomography (µCT) [30]. Some of these techniques have some disadvantages; radiography does not allow three-dimensional (3D) assessment of the minimum canal wall thickness [31]. SEM does not allow pre- and post-instrumentation comparison of the dentin thickness; but is an inherently invasive technique because of sample preparation and provides only two- dimensional images [32]. 

Replication of the internal canal anatomy by using impression materials and models are extremely technique sensitive [21]. Using the μCT is undoubtedly regarded as an excellent technique for experimental endodontology [33]. It has some disadvantages including high cost, not being readily available and being time-consuming in the reconstruction and measurement of each slice [34].

Since some of the techniques cause damage to tooth structure, utilizing a method with the least damage to the specimens is required. Application of CBCT in endodontics provides 3D images of the anatomic features and has been successfully used to evaluate the performance of endodontic rotary instruments on shaping the root canals [35, 36]. This technique provides accurate, reproducible evaluation of changes in anatomic structure of root canal before and after instrumentation without destruction of the specimens [37]. In this study, CBCT imaging permitted the reliable analysis of the changes in dentin thickness in coronal third of root canals after endodontic instrumentation.

Numerous variables were considered during the design of this study. Although the type of the instrument is of great importance, the result of the study by Kuttler *et al.* [38], indicated that pre-instrumentation dentinal thickness is the most important factor in determining the remaining canal wall thickness after preparation. No data was available in literature concerning the effect of RCP movement of WaveOne and its effect on dentin thickness in the DZ.

The results of the current study provides evidence on the lack of significant differences in the thickness of dentinal walls in the DZ area after instrumentation with WaveOne instruments in RCP or CCWR motions. Further research is recommended to compare WaveOne with other single-file systems in curved canals.

## Conclusion

Regardless of the motion type, preparation with the WaveOne file will reduce the thickness of remaining canal wall; however, there was no statistically significant difference between reverse continuous rotation and reciprocation of the instrument.
